# The Use of Opioids and Pain Medications in the Management of Postoperative Pain in Total Knee Arthroplasty Patients: A Retrospective Study

**DOI:** 10.1155/2022/7445144

**Published:** 2022-03-29

**Authors:** Ammar Almustafa, Asdghig Der-Boghossian, Abdel Majid Sheikh Taha

**Affiliations:** ^1^Department of Psychology, School of Social Sciences, Heriot-Watt University, Putrajaya, Malaysia; ^2^Division of Orthopaedic Surgery, Department of Surgery, Faculty of Medicine, American University of Beirut Medical Center, Beirut, Lebanon

## Abstract

**Purpose:**

The opioid crisis amplified the concern for the appropriate use of opioids. Our study aims to investigate the pain levels and amount of opiates needed during the first three days following total knee arthroplasty (TKA), whereby Drug Enforcement Administration (DEA) Schedule II oral opiates are not available.

**Methods:**

A year-long retrospective review of adult patients who underwent TKA was conducted. The postoperative pain scores and mean morphine equivalents (MME) were measured. These outcomes were assessed according to demographics, anesthesia, and analgesia used.

**Results:**

For our 78 patients, there was no statistical difference for stratification by baseline characteristics except in spinal anesthesia, which decreased pain on the first day. Conversely, MME increased to its significantly highest of 14.22 ± 29.58 mg on day 3. The effect was noted for patient-controlled analgesia where patients with intravenous analgesia received less opioid than those on epidural analgesia on postoperative day one.

**Conclusions:**

Using a similar regimen of analgesia, postoperative pain following TKA would be controlled by a relatively low amount of opioids by the third postoperative day. Spinal anesthesia and patient-controlled epidural analgesia were linked to better pain control and less opioid needed.

## 1. Introduction

Total knee arthroplasty (TKA) is a common procedure, usually used to treat knee osteoarthritis. Around 700000 and more than 100000 procedures are performed annually in the US and UK, respectively [1]. This number is expected to increase over the upcoming decades, projecting growth of 601% in procedures from 2005 to 2030 in the US [2].

TKA is a cost-effective procedure with a good patient satisfaction rate. Most studies reported a satisfaction rate of approximately 80%. Patients stated decreased pain levels, improved function, and better quality of life postoperatively. Patient satisfaction level was associated, among other factors, with less pain and improved functional status, while dissatisfaction was associated with a longer hospital stay [3]. An important component of healing following TKA is postoperative pain and its management that accelerates recovery and improves patient satisfaction [4].

Proper management of postoperative pain speeds patient's mobilization, decreases thromboembolic complications, improves main physiological functions, shortens the length of hospital stay, and improves patient's participation in physical therapy. Thus, optimal pain management helps in patient's wellbeing while reducing healthcare costs [5].

TKA usually has moderate to severe postoperative pain levels. The anesthetic-controlled pain score following TKA ranged from 2.7 to 7.64 on the visual analogue scale [4]. TKA pain management and anesthesia are highly diverse with many proposed multimodel regimens and are even expressed as follows: “No globally recognized, best proven, gold-standard analgesic treatment or intervention exists for TKA” [6]. The American Pain Society advises further research toward assessing and determining evidence-based practices in pain management [7]. A Presidential Commission was formed following the opioid crisis in the US with one of its goals to limit the prescription of opioids [8]. Unlike the US, other countries do not have readily available oral opioids outside the hospital setting. We want to assess pain management in a setting where pain medications of the Drug Enforcement Administration (DEA) Schedule II oral opiate group are not available to the patients, perioperatively. Thus, opiate use is limited to their intravenous forms and administered only to inpatients during the immediate postoperative period.

Our study evaluates the pain level and the amount of opioid consumed during the first three postoperative days following TKA and describes any differences in these outcomes by the various patient characteristics, in a setting where DEA Schedule II oral pain medication is not available.

## 2. Patients and Methods

We followed the retrospective observational study design. The institutional review board at our local institution approved the study.

The latest consecutive 100 adult patients who had undergone primary TKA during a year were identified from our institutional inpatient database using the appropriate Current Procedural Terminology (CPT) code of 27447. These surgeries were performed by multiple orthopedic surgeons; however, all followed institutionalized protocols. Tourniquets were used in all of the surgeries and anesthesia dosage followed per kg of weight guidelines.

The inclusion criteria consisted of any patient over 18 years old who had undergone primary TKA. The exclusion criteria included any patient who had undergone previous knee surgery, had diabetic neuropathy, had a history of chronic pain (diagnosed or recorded for more than 3 months unrelated to the knee), had any psychiatric or neurologic disease, or had a history of drug dependence. The excluded patients also included those who were taking any psychoactive drug, analgesics, or medication having an analgesic effect (steroid and anticonvulsants) for a period longer than 3 months, those who did not receive patient-controlled analgesia (PCA)/patient-controlled epidural analgesia (PCEA), and any patient who had more than 30% of the pain or pain medication data missing.

### 2.1. Data Collection

The collected data included patient characteristics (age, gender, smoking, education levels, and medical insurance status), surgery-related variables (diagnosis, laterality, and preoperative analgesia), pain levels, and details of the anesthetic and analgesics or any similar effect medication used during their admission for TKA both preoperatively and postoperatively.

The two main outcomes were pain score and amount of administered opioids.

The pain scores were reported using the numerical rating scale (NRS), where 0 indicates no pain and 10 indicates extreme pain. We derived the NRS scores for each day of the patients' hospital stay and calculated the mean NRS score for the first three days following TKA.

Similarly, we collected the amount of pain medication and opioids received by each patient for the first three days following TKA and calculated its correspondent morphine milligram equivalent (MME) in mg [9]. We measured and reported the mean of daily administered MME for each postoperative day. The PCA and PCEA were recorded as present or absent. The administration of PCA and PCEA followed the institutional protocol where morphine was administered at 2 mg/hour basal rate and a bolus of 2 ml with a lockout period of 10 minutes for PCA and Marcaine 0.1% mixed with fentanyl 2 ug/ml at 10 ml/hour basal rate and a 3 ml bolus with lockout period of 15 minutes for PCEA.

### 2.2. Statistical Analysis

The data were recorded, cleaned, and analyzed using IBM SPSS Statistics for Windows, version 25 (IBM Corp., Armonk, NY, USA). The mean and standard deviation (SD) was used for continuous variables; while frequency and percent were used for categorical data. The pain scores and MME distribution were not normally distributed; hence, nonparametric tests were performed. The daily values were compared using Friedman's two-way analysis of variance (ANOVA) among the three days and the Wilcoxon signed rank test between the two days. Furthermore, we stratified the pain scores and the MME levels by the patients' baseline variables and the means were compared using the Mann–Whitney *U*-test or Kruskal–Wallis test as appropriate. A confidence interval of 95% and a *p* value of <0.05 (with Bonferroni correction as needed) were used to indicate the statistically significant difference for all analyses.

## 3. Results

**Figure 1:**
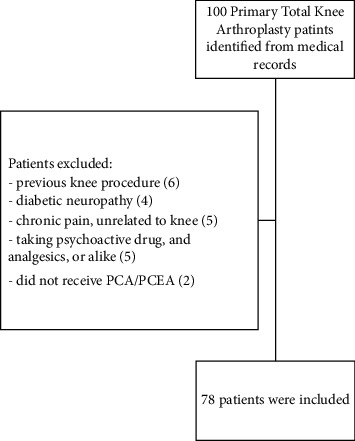
The flow diagram of the study patients' selection.

Seventy-eight TKA patients met the inclusion/exclusion criteria and were included (Figure 1). The majority of study patients were around 60 years old, females (74.4%), nonsmokers (76.9%), and had medical insurance coverage (85.7%).

Knee osteoarthritis was the indication for surgery in almost all patients. Most of the TKAs were unilateral (73%), did not receive preoperative analgesia (79.5%), and underwent either spinal, epidural, and/or general anesthesia. No local anesthetics were applied during any of the cases. During surgery, the mean duration of anesthesia was 253.77 ± 117.46 minutes. Postoperatively, 38.5% received PCA and 61.5% received PCEA for pain control (Table 1). All patients have received 1000 mg acetaminophen intravenously every 6 hours.

The mean daily pain score was the highest on the first postoperative day with a value of 2.10 ± 1.67 and decreased gradually in the following days (Table 2). A significant difference existed between the mean score of postoperative day 1 and that of day 3 (*p* < 0.01). Moreover, while the differences between preoperative versus postoperative days 1 or 2 pain scores were statistically significant with *p* < 0.01, the difference between preoperative and postoperative day 3 scores was nonsignificant with a Bonferroni-corrected *p* value of 0.10.

Alternatively, the maximum amount of MME was 81 mg on the postoperative day 1, 135 mg on day 2, and 148 mg on day 3. The daily MME was lowest on day 1 and reached its highest by postoperative day 3 (6.35 ± 14.79 mg vs. 14.22 ± 29.58 mg). However, the difference reached the border of statistical significance only for postoperative day 3 compared to postoperative day 1.

The stratification of pain score did not show any significant difference between genders, smoking status, education level, medical insurance, laterality of TKA, preoperative analgesia, preoperative pain, anesthesia types, and postoperative PCA/PCEA (Table 2). A significant decrease in mean pain score was noted only on the first day following TKA in those who received spinal anesthesia versus those who did not (*p* < 0.01).

Similarly, the stratification of MME by baseline variables did not show any significant difference except for PCA versus PCEA (Table 3). The mean MME levels were statistically higher in those receiving PCA compared to PCEA but only on postoperative day 1 (*p* < 0.01). On postoperative days 2 and 3, the difference disappeared. Some other variables showed a similar trend between day 1 and day 3, but did not reach a statistically significant difference in any postoperative day.

## 4. Discussion

Our study showed that postoperative pain following TKA and its control or opiate amounts are influenced by some baseline variables.

This study is not without limitations. The retrospective design of the study limited our access to standardized methods of outcome evaluations; however, the study institution does follow a specific protocol for pain management, which provided comparable and good-quality data. Also, the small sample size decreased the effect size of the study. The exact amounts of PCEA/PCA medication were not available, but its use was based on standardized institutional protocol. Otherwise, the administration of all other pain medications was detailed and accounted for. Additionally, there was a substantial overlap between anesthetics used by different patients with many receiving spinal, epidural, and general anesthetics in their management as this is the model used in the institution.

However, our sample was representative of the traditional population undergoing TKA in a specific region of the developing world. The majority of patients were females with a mean age of 58 and underwent unilateral TKA (Table 1). Based on registry data, most patients undergoing TKA were females. Although the mean age of surgery was previously reported to be 60 years and above, more recent data have shown an increase in the number of younger patients [1]. Better postoperative pain control leading to shorter recovery is even more vital in younger patients who are more functional and more active socially and economically.

In our study, the overall pain following TKA was mild and/or controlled by medications, but certain variables were noted which might affect the postoperative pain score (Table 2). A significantly lower pain score was noted for those who underwent spinal anesthesia versus whose anesthesia protocol excluded any. Spinal anesthesia has previously shown advantages versus general anesthesia for primary and revision TKA [10, 11]. Nevertheless, the anesthesia protocol for TKA is not well established yet [12]. Spinal anesthesia, alone or as part of multimodal anesthesia protocol, has shown lesser postoperative pain scores in other orthopedic surgeries [12]. This study supports the inclusion of spinal anesthesia in the anesthetic protocol of TKA if viewed from the pain control perspective.

Additionally, the pain scores started highest on postoperative day 1 and decreased gradually over postoperative days 2 and 3. Pain levels only reached the significant decline threshold by postoperative day 3 as compared to day 1. Moreover, the statistically and clinically significant difference in pain scores between the preoperative score and postoperative day 3 score disappeared, indicating complete control of surgical pain by day 3 following TKA.

One reason for the pain control might be the increased use of opioids by day 3, which was noted to be the highest on postoperative day 3 in our cohort (Table 3). Assuming the latter proposition, the MME of 14.22 ± 29.58 mg is sufficient to control the pain resulting from TKA by day 3 in the presence of PCA/PCEA, and a lesser dose of opioids should be sufficient to control pain in the first two days following TKA. Recently, Premkumar et al. [13] reported the mean amount of oral morphine equivalent consumed in the first six weeks following TKA to be 639.6 ± 323.7 mg, with a range of 20–1616 mg in the absence of PCA/PCEA. Despite variations in the pain management approach, this lowest range of opioid use (20 mg) seems comparable and only slightly higher than our mean of 14 mg for opioid use. Thus, analogous doses might be sufficient for pain control following TKA.

Those who received PCA required a much higher amount of opioids on postoperative day 1 than those who received PCEA, but this difference became nonsignificant in later days. Beilin et al. [14] have previously shown, as part of its secondary outcomes, that patients who underwent spinal-epidural anesthesia with PCEA had lower pain scores compared to those who underwent general anesthesia with PCA during the first twenty-four hours following TKA. Despite dampened and nonsignificant changes in pain scores between PCEA and PCA, a difference in opioid intake is evident. Thus, PCEA use following TKA will provide pain control with less opioid for the first day but not on postoperative days 2 and 3.

A similar demeanor was also noted across the postoperative days in those receiving spinal anesthesia, epidural anesthesia, or nerve block. Almost three times, the amount of administered opioid was needed on the first postoperative day in those not receiving spinal/epidural anesthesia or nerve block compared to those who did. However, this increase did not level up to a statistically significant difference.

In conclusion, postoperative pain and its control following TKA have multifactorial aspects that can affect the success of an opioid-sparing pain control regimen. The pain following TKA was the highest on the first postoperative day with a quick and substantial decrease in subsequent days. Our study revealed that morphine equivalent of around 14 mg is sufficient to control postoperative pain, in the presence of PCA/PCEA. This amount can be an anchor amount for future studies investigating opioid-sparing pain control regimens following TKA. In addition, the anesthesia choice and the presence of PCA or PCEA influence the pain levels during the first postoperative day but not afterward. Hence, spinal anesthesia and PCEA are better options in providing good control of short-term postoperative pain and administering less opiate in TKA patients. Furthermore, thorough studies are needed to evaluate the effect of various variables on postoperative pain and its control following TKA.

## Figures and Tables

**Table 1:** The baseline variables of the patients who underwent total knee arthroplasty (TKA).

Baseline variables	*n*	Frequency (%)
Age^a^	77	67.51 ± 6.66
Gender (female)	78	58 (74.4%)
Weight^a^	78	88.97 ± 25.36
Smoking (yes)	78	18 (23.1%)
Education	78	
Less than high school		15 (24.2%)
High school		30 (48.4%)
College or higher		17 (27.4%)
Medical insurance	78	
No/self-paying		11 (14.3%)
Yes		66 (85.7%)
Private insurance		46 (59.7%)
State coverage/more than 1 coverage		20 (26%)
Laterality of TKA (unilateral)	78	54 (69.2%)
Preoperative analgesia (yes)	78	16 (20.5%)
Preoperative pain^a^	72	0.58 ± 1.84
Anesthesia approach		
Spinal (yes)	78	53 (67.9%)
Epidural (yes)	78	49 (62.8%)
General (yes)	78	47 (60.3%)
Nerve block (yes)	78	5 (6.4%)
Analgesia method	78	
Patient-controlled analgesia (PCA)		30 (38.5%)
Patient-controlled epidural analgesia (PCEA)		48 (61.5%)
Duration of anesthesia induction^a^	78	253.77 ± 117.46

^a^Mean ± standard deviation is reported instead of frequency. *n*, the sample size

**Table 2:** The overall and stratified means and standard deviation (SD) of the daily pain scores of the postoperative days one, two, and three.

Pain score	Postoperative day 1	*P* value	Postoperative day 2	*P* value	Postoperative day 3	*P* value
Overall, mean ± SD (*n*)	2.10 ± 1.67 (76)	<0.01^a^	1.68 ± 2.28 (56)	0.13^b^	1.26 ± 2.12 (51)	<0.01^a^
Per baseline characteristics						
Gender						
Male	2.10 ± 1.61	0.68	1.38 ± 2.42	0.32	0.93 ± 1.56	0.53
Female	2.10 ± 1.71		1.80 ± 2.23		1.38 ± 2.30	
Smoking status						
No	2.15 ± 1.75	0.81	1.65 ± 2.42	0.26	1.12 ± 1.93	0.28
Yes	1.93 ± 1.43		1.76 ± 1.72		1.82 ± 2.81	
Education level						
Less than high school	2.22 ± 1.45	0.68	0.56 ± 0.92	0.16	1.61 ± 2.42	0.87
High school	2.13 ± 1.73		1.76 ± 2.52		1.04 ± 1.87	
College or higher	1.68 ± 1.45		2.10 ± 2.23		0.98 ± 1.61	
Medical insurance						
No/self	1.40 ± 1.11	0.16	0.79 ± 1.35	0.25	2.00 ± 3.52	0.70
Yes	2.23 ± 1.74		1.80 ± 2.36	0.36	1.16 ± 1.89	
Laterality of TKA						
Unilateral	2.00 ± 1.80	0.11	1.56 ± 2.10	0.62	1.25 ± 2.22	0.86
Bilateral	2.54 ± 1.30		1.75 ± 2.76		1.27 ± 2.09	
Preoperative analgesia						
No	2.00 ± 1.55	0.51	1.82 ± 2.35	0.15	1.15 ± 2.09	0.47
Yes	2.52 ± 2.10		1.00 ± 1.87		2.08 ± 2.31	
Anesthesia approach						
Spinal						
No	2.88 ± 1.83	<0.01	2.09 ± 2.31	0.24	1.57 ± 2.24	0.36
Yes	1.72 ± 1.46		1.43 ± 2.25		1.00 ± 2.01	
Epidural						
No	2.53 ± 1.90	0.11	1.72 ± 2.24	0.94	1.32 ± 1.79	0.39
Yes	1.83 ± 1.47		1.65 ± 2.33		1.21 ± 2.40	
General						
No	1.67 ± 1.38	0.09	1.38 ± 2.19	0.53	1.34 ± 2.33	0.77
Yes	2.38 ± 1.80		1.85 ± 2.34		1.21 ± 2.01	
Nerve block						
No	2.10 ± 1.62	0.78	1.72 ± 2.30	0.49	1.24 ± 2.11	0.95
Yes	2.12 ± 2.84		0.33 ± 0.47		1.50 ± 2.60	
Patient-controlled analgesia						
Patient-controlled analgesia (PCA)	2.25 ± 1.98	0.80	1.29 ± 2.17	0.14	1.38 ± 1.90	0.37
Patient-controlled epidural analgesia (PCEA)	2.01 ± 1.46		1.95 ± 2.35		1.14 ± 2.32	

*n*, sample size. ^a^Related-samples Friedman's two-way analysis of variance across all groups comparison. ^b^Related-samples Wilcoxon signed rank test as compared to postoperative day 1. *P* value = 0.05 for comparison of pain score between postoperative day 2 and day 3. *P* value <0.05 indicates significance.

**Table 3:** The overall and stratified means and standard deviation (SD) of the daily morphine equivalent administered on the postoperative days one, two, and three.

Mean morphine equivalent	Postoperative day 1	*P* value	Postoperative day 2	*P* value	Postoperative day 3	*P* value
Overall, mean ± SD (*n*)	6.35 ± 14.79 (78)	0.71^a^	10.42 ± 27.05 (78)	0.65^b^	14.22 ± 29.58 (78)	0.05^b^
Per baseline characteristics						
Gender						
Male	10.20 ± 18.35	0.22	8.55 ± 22.22	0.65	19.85 ± 19.85	0.06
Female	5.02 ± 13.28		11.07 ± 28.67		12.28 ± 29.91	
Smoking status						
No	6.15 ± 15.76	0.44	12.30 ± 29.88	0.16	15.95 ± 32.27	0.53
Yes	7.00 ± 11.32		4.17 ± 12.65		8.44 ± 17.39	
Education level						
Less than high school	11.20 ± 21.41	0.39	22.60 ± 41.40	0.40	15.53 ± 33.79	0.78
High school	3.80 ± 8.88		7.20 ± 25.09		18.87 ± 35.04	
College or higher	10.94 ± 20.45		9.71 ± 24.41		18.23 ± 26.90	
Medical insurance						
No/self	11.73 ± 23.02	0.77	10.91 ± 27.73	0.70	17.27 ± 34.08	0.91
Yes	5.54 ± 13.09		10.50 ± 27.32		13.92 ± 29.20	
Laterality of TKA						
Unilateral	8.00 ± 16.63	0.13	9.83 ± 26.43	0.79	16.74 ± 33.52	0.66
Bilateral	3.15 ± 9.41		12.60 ± 31.19		9.25 ± 18.27	
Preoperative analgesia						
No	6.97 ± 15.82	0.70	9.08 ± 25.12	0.18	14.34 ± 30.58	0.95
Yes	3.94 ± 9.90		15.62 ± 33.97		13.75 ± 26.21	
Anesthesia approach						
Spinal						
No	8.04 ± 18.24	0.51	13.08 ± 35.47	0.95	14.72 ± 36.57	0.46
Yes	5.55 ± 12.98		9.17 ± 22.30		13.98 ± 26.04	
Epidural						
No	11.48 ± 21.35	0.06	20.52 ± 40.73	0.08	14.90 ± 31.67	0.80
Yes	3.31 ± 7.73		4.45 ± 10.38		13.82 ± 28.60	
General						
No	6.00 ± 14.70	0.85	11.55 ± 27.71	0.94	16.77 ± 29.58	0.23
Yes	6.57 ± 15.01		9.68 ± 26.88		12.53 ± 29.77	
Nerve block						
No	6.66 ± 15.22	0.71	10.10 ± 27.73	0.18	14.29 ± 29.78	0.86
Yes	1.80 ± 4.02		15.20 ± 14.60		13.20 ± 29.52	
Analgesia method						
Patient-controlled analgesia (PCA)	12.00 ± 20.77	<0.01	18.60 ± 40.50	0.78	12.80 ± 29.01	0.61
Patient-controlled epidural analgesia (PCEA)	2.81 ± 7.69		5.31 ± 10.83		15.10 ± 30.19	

*n*, sample size. ^a^Related-samples Friedman's two-way analysis of variance across all groups comparison. ^b^Related-samples Wilcoxon signed rank test as compared to postoperative day 1. *P* value = 0.52 for comparison of mean morphine equivalent between postoperative day 2 and day 3. *P* value <0.05 indicates significance.

## Data Availability

The data used to support the findings of this study are available from the corresponding author upon request.
